# Proteoglycan-Dependent Endo-Lysosomal Fusion Affects Intracellular Survival of *Salmonella* Typhimurium in Epithelial Cells

**DOI:** 10.3389/fimmu.2020.00731

**Published:** 2020-04-29

**Authors:** Alibek Galeev, Abdulhadi Suwandi, Hans Bakker, Ade Oktiviyari, Françoise H. Routier, Lena Krone, Michael Hensel, Guntram A. Grassl

**Affiliations:** ^1^Institute of Medical Microbiology and Hospital Epidemiology, Hannover Medical School and German Center for Infection Research (DZIF), Hanover, Germany; ^2^Institute of Clinical Biochemistry, Hannover Medical School, Hanover, Germany; ^3^Division of Microbiology, University of Osnabrück, Osnabrück, Germany

**Keywords:** *Salmonella*, proteoglycans, glycosaminoglycans, xylosyltransferase, PIKfyve, gentamicin

## Abstract

Proteoglycans (PGs) are glycoconjugates which are predominately expressed on cell surfaces and consist of glycosaminoglycans (GAGs) linked to a core protein. An initial step of GAGs assembly is governed by the β-D-xylosyltransferase enzymes encoded in mammals by the *XylT1/XylT2* genes. PGs are essential for the interaction of a cell with other cells as well as with the extracellular matrix. A number of studies highlighted a role of PGs in bacterial adhesion, invasion, and immune response. In this work, we investigated a role of PGs in *Salmonella enterica* serovar Typhimurium (*S*. Typhimurium) infection of epithelial cells. Gentamicin protection and chloroquine resistance assays were applied to assess invasion and replication of *S*. Typhimurium in wild-type and xylosyltransferase-deficient (Δ*XylT2*) Chinese hamster ovary (CHO) cells lacking PGs. We found that *S*. Typhimurium adheres to and invades CHO WT and CHO Δ*XylT2* cells at comparable levels. However, 24 h after infection, proteoglycan-deficient CHO Δ*XylT2* cells are significantly less colonized by *S*. Typhimurium compared to CHO WT cells. This proteoglycan-dependent phenotype could be rescued by addition of PGs to the cell culture medium, as well as by complementation of the *XylT2* gene. Chloroquine resistance assay and immunostaining revealed that in the absence of PGs, significantly less bacteria are associated with *Salmonella*-containing vacuoles (SCVs) due to a re-distribution of endocytosed gentamicin. Inhibition of endo-lysosomal fusion by a specific inhibitor of phosphatidylinositol phosphate kinase PIKfyve significantly increased *S*. Typhimurium burden in CHO Δ*XylT2* cells demonstrating an important role of PGs for PIKfyve dependent vesicle fusion which is modulated by *Salmonella* to establish infection. Overall, our results demonstrate that PGs influence survival of intracellular *Salmonella* in epithelial cells via modulation of PIKfyve-dependent endo-lysosomal fusion.

## Introduction

Proteoglycans (PGs) are heavily glycosylated proteins facilitating cell-matrix and cell-cell interactions and are also playing an important role in bacterial adhesion, invasion, and immune response (1). All PGs consist of a core protein substituted with glycosaminoglycans (GAGs) – long linear polysaccharides comprised of repeating disaccharide units. Based on the structure of the disaccharide unit, GAGs are divided into four distinct families: heparan sulfate (HS)/heparin, chondroitin/dermatan sulfate, keratan sulfate, and hyaluronan (2). The biosynthesis of the first two GAGs is initiated by the assembly of the tetrasaccharide linker D-GlcA-β1-3-Gal-β1-3-Gal-β1-4-Xyl-β-Ser. The initial, rate-limiting step is the transfer of Xyl from UDP-D-α-xylose to serine moieties of the core protein and is catalyzed by the isoenzymes β-D-xylosyltransferase-I and -II (EC 2.4.2.26) (3) encoded in humans by the *XYLT1* and *XYLT2* genes, respectively.

In the past years, a number of studies highlighted an importance of PGs in bacterial pathogenesis. Proteoglycan-mediated adhesion and invasion has been previously reported for various gram-positive and gram-negative bacteria, including *Listeria monocytogenes* (4), *Neisseria gonorrhoeae* (5), *Borrelia burgdorferi* (6), and *Salmonella enterica* serovar Typhimurium (*S*. Typhimurium) (7). *S*. Typhimurium is a successful food-borne pathogen able to colonize the human gastrointestinal tract and to cause severe diarrhea. *S*. Typhimurium manipulates actin and membrane trafficking pathways of epithelial cells initiating entry via actin-mediated macropinocytosis (8). A hallmark of *Salmonella* infection is an extensive alteration of the endo-lysosomal system and phosphoinositide metabolism of the host (9). After invasion, *S*. Typhimurium translocates effectors through a type 3 secretion system (T3SS) encoded by genes in *Salmonella* pathogenicity island 2 (SPI2) into the host cytoplasm in order to establish a replicative niche called the *Salmonella*-containing vacuole (SCV). At later stages of infection, mature SCVs acquire late endosomal markers, such as LAMP1, and fuse with the late endosomes and other organelles (ER, Golgi apparatus) forming an extensive network of tubules called *Salmonella*-induced filaments (SIFs) (10, 11). Previous studies identified that SIF formation by *S*. Typhimurium is dependent on various host factors, including the lysosomal glycoproteins LAMPs (12), vacuolar vATPase (13), the late endosomal small GTPase Rab7 (14), and secretory carrier membrane proteins (SCAMPs) (15). Moreover, there is a growing body of evidence suggesting that *Salmonella* interferes with the exocytic transport machinery and with secretory pathways (16, 17).

Phosphatidylinositol (PI) belongs to the class of the phosphatidylglycerides – glycerol-based phospholipids which are a major component of biological cell membranes. Phosphorylated forms of PI (called phosphoinositides) play important roles in cell signaling, cell growth and death, and membrane trafficking (18). For example, the monophosphorylated phosphatidylinositol-3-phosphate (PtdIns3P) orchestrates recruitment and membrane association of early endosomal proteins EEA1 and Rab5 (19). Furthermore, it was demonstrated that phosphatidylinositol-3,5-bisphosphate (PtdIns(3,5)P_2_) regulates endosomal fission and fusion, as well as multivesicular body (MVB) formation and detachment (20). PIKfyve is a lipid kinase that converts PtdIns3P into PtdIns(3,5)P_2_ in the endocytic microdomains of mammalian cells (21). While it is known that *Salmonella* can modulate phosphoinositide pathways in host cells (22, 23), limited knowledge exists on possible interactions of phosphoinositides with PGs.

To investigate the contribution of surface and intracellular PGs to *Salmonella* infection we utilized a proteoglycan-deficient Chinese hamster ovary (CHO) cell line (3). We demonstrate that absence of PGs in epithelial CHO cells results in an altered PIKfyve-dependent endo-lysosomal trafficking affecting intracellular *Salmonella* survival.

## Materials and Methods

### Cell Lines

Chinese hamster ovary (CHO) CHO-K1 WT and CHO-K1 pgsA745 (aka Δ*XylT2*, referred to as Δ*XylT*) (3) cells were routinely cultured in DMEM/F-12 GlutaMAX growth medium (Life Technologies) supplemented with 10% (v/v) fetal bovine serum (Biochrom).

### CHO Δ*XylT* Cell Line Complementation

CHO Δ*XylT* mutant was complemented with a plasmid expressing human *XYLT2* as described (24). Complementation of a G418-selected clone was confirmed by flow cytometry using the heparan sulfate-specific phage display antibody AO4B08 (25). The overlaid histograms with the peak heights were normalized to mode (% of Max). The data were analyzed using FlowJo v.10 software (TreeStar).

### Bacteria and Growth Conditions

*Salmonella enterica* serovar Typhimurium (*S*. Typhimurium) 14028s (26), *S*. Typhimurium SL1344 WT (27), *S*. Typhimurium SL1344 eGFP (pFPV25.1) (28), *S*. Typhimurium SL1344 Δ*ssaR* (29), and *S*. Typhimurium SL1344 Δ*sifA* (30) were grown overnight at 37°C with shaking in lysogeny broth (LB) supplemented with 100 μg/mL streptomycin, 100 μg/mL ampicillin, or 50 μg/mL kanamycin, when appropriate. The reporter strain *S*. Typhimurium SL1344 p4889 (31) was grown in presence of carbenicillin 50 μg/mL. *Listeria monocytogenes* EGD strain (32) was grown at 37°C in Brain Hearth Infusion (BHI) broth. For infection, overnight cultures of bacteria were sub-cultured and grown for 3 h at 37°C to mid-log phase.

### Generation of Acid Shock Reporter Plasmid

The acid shock-responsive promoter of *asr* was used to control expression of sfGFP. A dual fluorescence reporter was generated based on p4889, and P*_*uhpT*_* in p4889 was replaced by P*_*asr*_* by Gibson assembly (GA) cloning. Primers Vf-p4889 and Vr-p4889 were used to PCR amplify the vector backbone of p4889, and 1f p4889-Pasr and 1r Pasr-sfGFP were used to amplify the P*_*asr*_* region from genomic DNA of *S*. Typhimurium. GA resulted in plasmid p5386 that was confirmed by DNA sequencing, and functional analyses of response of sfGFP expression upon acid shock exposure in synthetic media buffered to various pH.

### Gentamicin Protection Assay

CHO WT, CHO Δ*XylT*, or complemented CHO Δ*XylT* cells (CHO cX) were seeded in 24-well plates (10^5^ cells/well) and incubated overnight in 5% CO_2_ at 37°C. The next day, cells were infected with either *Listeria monocytogenes* EGD, or with different *Salmonella* strains at MOI 10, 50, or 100 (as indicated). For quantification of adherent bacteria, 30 min (or 60 min for *Listeria*) post infection (p.i.), cells were washed 3 times with PBS and lysed in PBS containing 1% (v/v) Triton X-100 and 0.1% (v/v) sodium dodecyl sulfate (SDS). The cell lysates were then serially diluted in PBS and plated on LB agar or on BHI agar for colony-forming unit (CFU) count. For later time points, upon washing, culture medium was replaced with a medium supplemented with 100 μg/mL gentamicin (Sigma) to kill extracellular bacteria. The number of invaded bacteria was determined by plating the cells lysates 1.5 h p.i. (2.5 h p.i. for *Listeria*). Medium was replaced by medium containing the indicated concentrations of gentamicin and bacterial intracellular survival or replication was assessed 4 and 24 h p.i.

### Gene Expression Analysis

Total RNA was extracted from CHO cells using the High Pure RNA Isolation Kit (Roche) following the manufacturer’s guidelines. Reverse transcription of 1 μg RNA of each sample was done with the High Capacity cDNA Reverse Transcription Kit (Applied Biosystems). Gene expression was assessed by qPCR using the Power SYBR^®^ Green PCR Master Mix (Applied Biosystems) using gene specific primers (Supplementary Table S1). Relative gene expression was calculated by the ΔΔCt method (33) and normalized to *Gapdh* and *Rps9* housekeeping genes.

### Gentamicin ELISA

To determine levels of intracellular gentamicin, CHO WT and CHO Δ*XylT* cells were washed four times with PBS and lysed. The concentration of gentamicin in cell lysates was measured using the GEN ELISA Kit (Cusabio) according to the manufacturer’s protocol.

### Gentamicin Cy3 Conjugation and Cell Labeling Experiments

Gentamicin sulfate salt (Sigma-Aldrich) was mixed with the Sulfo-Cyanine3 NHS ester (Lumiprobe) in 50:1 molar ratio and incubated for 1 h at room temperature. The conjugate (Gen-Cy3) was isolated by reversed-phase chromatography (column C18), aliquoted, dried, and stored in the dark at -20°C. Prior to usage, the conjugate was resuspended in sterile water, absorbance at 548 nm was measured, and a concentration was calculated using the molar attenuation coefficient of the Sulfo-Cyanine3 NHS ester. In the cell labeling experiments, gentamicin sulfate used in a protection assay was replaced with Cy3-conjugated gentamicin (GEN-Cy3) at the indicated concentrations. CHO WT and CHO Δ*XylT* cells incubated with GEN-Cy3 were fixed at different time points post infection.

CHO WT and CHO Δ*XylT* cells were seeded on coverslips and then infected with *S*. Typhimurium eGFP at an MOI of 50. Upon bacterial invasion, CHO cells were incubated for 2, 7, or 24 h in presence of 50 nM Lysotracker Red DND-99 (Sigma-Aldrich). Then, CHO cells were extensively washed with PBS, fixed with 4% paraformaldehyde (PFA) and stained with 4′,6-diamidino-2-phenylindole (DAPI) (Invitrogen) (1:1000) to visualize nuclei. Images were recorded on a Zeiss Apotome.2 microscope using AxioVision 4.9.1 software (Zeiss).

### Immunocytochemistry

CHO WT and CHO Δ*XylT* cells were seeded on coverslips in 24 well plates, fixed with 4% PFA, washed 3 times with PBS, and permeabilized with Triton-X100 (0.1%). Unspecific binding was blocked using 2% normal goat serum (NGS), cells were then incubated with the AO4B08 antibody (25) (1:100) recognizing both heparin and HS. Infected cells were additionally stained with rabbit anti-*Salmonella* antibody (1:100). Upon washing, bound AO4B08 antibodies were detected by incubation with mouse anti-VSV tag IgG antibody P5D5 (1:400), followed by Alexa 488-conjugated goat anti-mouse IgG (1:1000) and Alexa 568-conjugated goat anti-rabbit IgG (1:1000) (Thermo Fisher Scientific). Phalloidin-iFluor 647 (1:1000) (Abcam) and DAPI (Invitrogen) were applied to visualize F-actin and nuclei, respectively. For a list of antibodies used see Supplementary Table S2.

### Chloroquine Resistance Assay

To determine the number of cytosolic *S*. Typhimurium within the CHO cells, chloroquine (CHQ) resistance assay was performed as described (34). Briefly, CHO WT and CHO Δ*XylT* cells were infected as described above. 24 h p.i., the cells were incubated for 1 h in the presence of CHQ (400 μM) and gentamicin (cytosolic bacteria) or with gentamicin only (total intracellular bacteria). CHO cells were then lysed, and serial dilutions plated on LB agar plates.

### Infection With *S*. Typhimurium Reporter Strains

CHO WT and CHO Δ*XylT* cells seeded on coverslips were infected with *S*. Typhimurium p4889 reporter strain at MOI 100. Upon bacterial invasion, CHO medium was supplemented with 100 μg/mL gentamicin. 24 h p.i., the infected cells and uninfected controls were fixed with 4% PFA, and then incubated with DAPI (1:1000). Images were recorded with a Zeiss Apotome.2 microscope using AxioVision 4.9.1 software (Zeiss). Bacteria in cytoplasm and in *Salmonella*-containing vacuole were enumerated in 20 random fields of view (FOV) using the Fiji software (35).

To test exposure of intracellular bacteria to acidic endosomal environments, CHO WT and CHO Δ*XylT* were infected with *S.* Typhimurium harboring p5386 at MOI of 10. Cells were incubated with or without gentamicin as indicated. CHO cells were detached 2 h after infection, chloramphenicol was added in a final concentration of 200 μg/mL to stop further bacterial protein biosynthesis, and incubation was continued at 4°C in order to allow full maturation of all synthesized sfGFP molecules. To control the effect of host cell endosomal acidification on intracellular *S*. Typhimurium, acidification was abrogated by vATPase inhibitor bafilomycin added after infection in a final concentration of 100 nM. For quantification, at least 50,000 CHO cells were analyzed by flow cytometry on an Attune NxT (Thermo Fisher) instrument. Cells were gated for DsRed-positive, i.e., *S*. Typhimurium-infected population, and sfGFP fluorescence of this population was determined as proxy for the level of acidification.

### Transfection

10^6^ CHO WT and CHO Δ*XylT* cells were resuspended in 100 μl Nucleofector Solution and mixed with 5 μg of p4605 plasmid encoding human ARL8B (ADP Ribosylation Factor Like GTPase 8B) (36) and transfected using the Nucleofector Program U-027 (Lonza). Immediately after transfection, cells were resuspended in CHO medium, seeded onto cover slips in 24 well plates, and incubated for at least 18 h prior to infection.

### Antibody Uptake Assay

CHO WT and CHO Δ*XylT* cells seeded on coverslips were infected with *S*. Typhimurium WT or with *S*. Typhimurium WT eGFP at an MOI of 50. 1.5 h p.i., medium was replaced with medium containing 10 μg/mL gentamicin and rabbit anti-*Salmonella* antibody (Difco). 24 h p.i., cells were fixed with 4% PFA, and bacteria were stained with the Alexa 546-conjugated donkey anti-rabbit IgG. In case of WT bacteria, samples were additionally stained with mouse anti-*Salmonella* antibody (Meridian Life Science) and Alexa 488-conjugated donkey anti-mouse IgG secondary antibody (Thermo Fisher Scientific). Bacteria were enumerated in 20 random FOVs.

### Endo-Lysosomal Fusion Assay

CHO WT and CHO Δ*XylT* cells were seeded on coverslips for pulse-chase experiments. In brief, CHO cells were first incubated with dextran-Alexa568 (10,000 MW, 0.4 mg/mL) (Invitrogen) for 4 h, washed and incubated in dextran-free CHO medium for 18 h. Cells were then pulsed with dextran-Alexa488 (10,000 MW, 0.4 mg/mL) for 10 min, washed and incubated in CHO medium for 30 min. CHO WT and CHO Δ*XylT* cells were fixed with 4% PFA, and stained with phalloidin-iFluor 647 Reagent (1:1000) (Abcam) and DAPI. Images of 20 random FOVs were acquired on Zeiss Apotome.2 microscope with 63 × oil immersion objective using AxioVision 4.9.1 software (Zeiss). Spatial resolution of images was 9.7674 pixels per micron, pixel size: 0.1024 × 0.1024 micron^2^. Endo-lysosomal fusion was scored by quantifying co-localization between the two labeled dextrans using ImageJ version 1.52e and JACoP plugin for pixel intensity spatial correlation analysis (37). Pearson’s correlation coefficient and Manders split coefficients (M1 and M2, thresholds set manually for both channels) were calculated.

### Cytotoxicity Assay

CHO cells were infected with *S.* Typhimurium WT strain as described previously. 24 h p.i., supernatants were collected and an activity of the lactate dehydrogenase (LDH) was measured using the Pierce LDH Cytotoxicity Assay Kit (Thermo Fisher Scientific) following the manufacturer’s instructions.

### Statistical Analysis

Data were analyzed using Prism V7.0d software (GraphPad). Statistical analysis was done using one-way analysis of variance (ANOVA) followed by Tukey’s multiple comparison test, or Dunnett’s multiple comparison test, the Kruskal-Wallis test followed by Dunn’s multiple comparison test, unpaired two-tailed t-test, or Wilcoxon-Mann-Whitney test as indicated. The results were considered statistically significant when *p*-values were smaller than 0.033. Graphs display the mean values ± SD and represent three independent biological repetitions unless stated otherwise.

## Results

### Proteoglycans Are Crucial for *Salmonella* Survival Within CHO Cells

**FIGURE 1:**
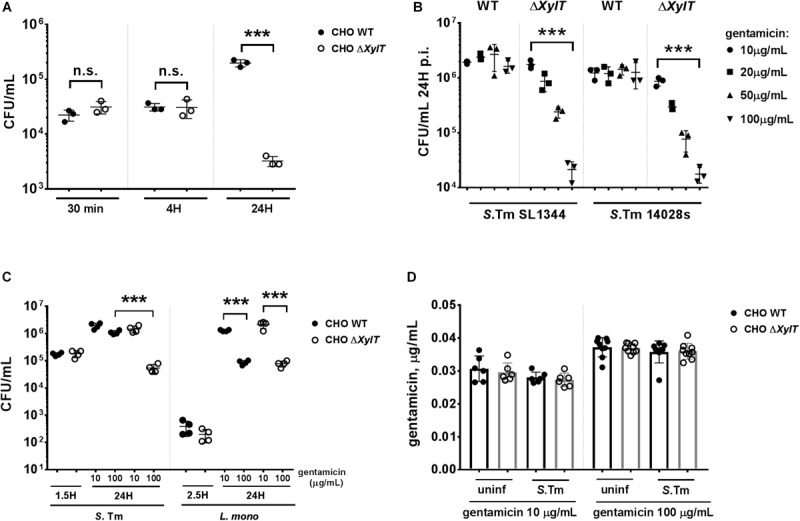
Proteoglycans influence survival of intracellular *S.* Typhimurium. **(A)** CHO WT and CHO Δ*XylT* cells were infected with *S*. Typhimurium WT at MOI 10 for 30 min, washed three times with PBS and then lysed to assess adherence. Remaining wells were incubated for 1 h with 100 μg/mL gentamicin to kill extracellular bacteria, and then either lysed immediately to evaluate invasion or at various time points post infection (p.i.) following incubation with media supplemented with 100 μg/mL gentamicin, as indicated. Lysates were collected, serially diluted and plated on agar plates. CHO Δ*XylT* (open circles), CHO WT cells (closed circles). **(B)** CHO WT and CHO Δ*XylT* cells were infected with *S*. Typhimurium SL1344 or with *S*. Typhimurium 14028s at MOI 50. Inhibition of bacterial growth (of both strains) in CHO Δ*XylT* cells was dependent on the gentamicin concentration used. **(C)** CHO WT and CHO Δ*XylT* cells were infected with either *S*. Typhimurium WT for 30 min., or with *L*. *monocytogenes* for 1 h (MOI 50) followed by treatment with 100 μg/mL gentamicin for 60 min. At 1.5 h (*Salmonella*) or 2.5 h (*Listeria*) medium was replaced with medium containing the indicated concentration of gentamicin. **(D)** Total cell lysates of the CHO WT and CHO Δ*XylT* cells at 24 h p.i. (non-infected, or infected with *S*. Typhimurium WT) incubated with 10 mg/mL or 100 μg/mL gentamicin were collected and levels of the intracellular gentamicin were measured by ELISA. Data points, means and SD of representative results of three independent experiments are depicted. One-way ANOVA with Tukey’s multiple comparison test, n.s. (not significant), ***p < 0.001.

Proteoglycans were shown to contribute to *Salmonella* invasion via interaction with the bacterial adhesin PagN (7). However, PagN is only expressed under intracellular (SPI2)-inducing conditions and when PagN is not expressed, invasion of host cells is PG-independent. Furthermore, it is not known if PGs are important for intracellular survival or replication. To test this, CHO WT and proteoglycan-deficient CHO Δ*XylT* cells were infected with *S.* Typhimurium WT strains. Bacterial adhesion (30 min p.i), invasion (1.5 h p.i), and early replication (4 h p.i) were comparable between CHO WT and CHO Δ*XylT* cells. However, 24 h p.i., we detected a significant reduction of intracellular bacteria in CHO Δ*XylT* compared to CHO WT cells incubated in presence of 100 μg/mL gentamicin (Figure 1A). Gentamicin-mediated killing of *S.* Typhimurium 14028S and SL1344 strains CHO Δ*XylT* cells was dose-dependent (Figure 1B). In contrast, when ampicillin was applied instead of gentamicin, a dose-dependent reduction of bacterial intracellular numbers was detected in the both CHO WT and CHO Δ*XylT* cells infected with *S.* Typhimurium 14028S at 24 p.i. (Supplementary Figure S1). To determine whether the effect on intracellular survival is *Salmonella*-specific, we infected CHO WT and CHO Δ*XylT* cells with another intracellular pathogen, *Listeria monocytogenes*. In contrast to *Salmonella*, reduction of intracellular *Listeria* was dependent on the gentamicin dose but not dependent on the presence of proteoglycans (Figure 1C).

Next, we asked whether proteoglycan can affect the uptake of gentamicin into CHO cells as increased uptake of gentamicin by CHO Δ*XylT* cells could contribute to increased killing of intracellular *Salmonella*. CHO cells were infected with *S.* Typhimurium WT as described above and intracellular gentamicin concentrations were measured by ELISA. No differences in intracellular gentamicin levels were detected between CHO WT and CHO Δ*XylT* cells, or between uninfected and infected cells (Figure 1D). Previous studies emphasized a role of the transient receptor potential channels (Trpv), Trpv1 (38) and Trpv4 (39), and the multidrug resistance protein 2 (Mrp2 or Abcc2) (40) in the cellular uptake and transport of gentamicin, respectively. Expression of *Trpv1*, *Trpv4*, and *Mrp2* genes was comparable in non-infected and infected CHO WT and CHO Δ*XylT* cells (Supplementary Figures S2A–C). Collectively, these findings indicate that a lack of proteoglycans in CHO cells does not affect active or passive gentamicin uptake.

**FIGURE 2:**
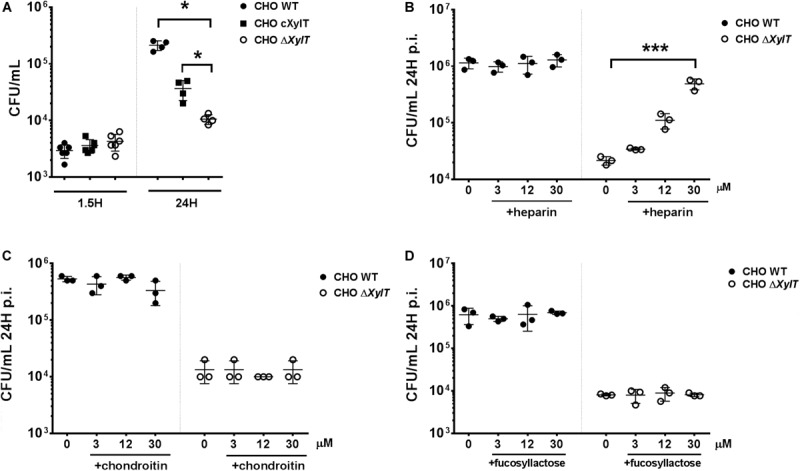
Proteoglycan-dependent phenotype can be rescued by an addition of the external GAGs as well as by *XylT2* gene complementation. CHO WT and CHO Δ*XylT* cells were infected with *S*. Typhimurium WT and incubated for 24 h with 100 μg/mL gentamicin or lysed immediately to evaluate invasion. **(A)** Infection of complemented of CHO Δ*XylT* cells (indicated as cXylT, in black squares) resulted in significantly higher numbers of intracellular *Salmonella* in comparison to CHO Δ*XylT* cells. Mann–Whitney *U* test, *p < 0.033. CHO WT and CHO Δ*XylT* cells were infected with *S*. Typhimurium WT at MOI 50. Gentamicin (100 μg/mL) was added 30 min p.i. Heparin sodium salt **(B)**, chondroitin sulfate **(C)** or 2′-fucosyllactose **(D)** was added at indicated concentrations 1.5 h p.i. Data points, means and SD of representative results of three independent experiments are depicted. One-way ANOVA with Dunnett’s multiple comparison test, not significant differences are not indicated, ***p < 0.001.

To verify that the observed phenotype is indeed due to the proteoglycan deficiency, we complemented the CHO Δ*XylT* cells with the human *XYLT2* gene. When compared to the proteoglycan-deficient CHO Δ*XylT* cells, complemented CHO c*XylT* cells harbored similar levels of *Salmonella* after invasion (at 1.5 h p.i.), but significantly more bacteria at 24 h p.i. (Figure 2A). However, while compared to CHO WT cells, complemented CHO c*XylT* cells still had lower *S.* Typhimurium loads 24 h p.i., which correlated with lower amounts of proteoglycans present on CHO c*XylT* cells, as assessed by flow cytometry (Supplementary Figure S3A), indicating only partial complementation of PGs. Next, we tested if addition of proteoglycans to the medium could also complement *Salmonella* survival in CHO Δ*XylT* cells. Addition of heparin (a structural analog of heparan sulfate) to the medium increased bacterial survival in CHO Δ*XylT* cells in a dose-dependent manner, but did not affect *Salmonella* survival in CHO WT cells (Figure 2B). In contrast, addition of equimolar amounts of chondroitin sulfate A (Figure 2C) or 2-fucosyllactose (Figure 2D) did not affect intracellular bacterial numbers in either CHO cell line. Notably, heparin did not support or inhibit growth of *S.* Typhimurium in LB medium and did not affect killing of *Salmonella* in LB broth supplemented with 100 μg/mL gentamicin (Supplementary Figure S3B). To summarize, these results indicate that host proteoglycans are important for the survival of *Salmonella* in epithelial cells when gentamicin is added to the tissue culture medium.

### CHO Cells Lacking Proteoglycans Display a Lower Abundance of SCV-Associated *Salmonella*

**FIGURE 3:**
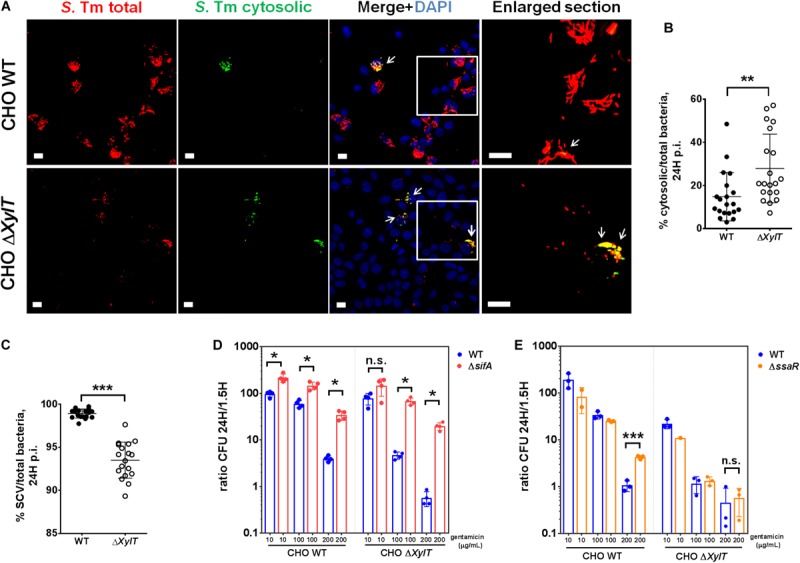
*Salmonella* in SCV, but not cytosolic bacteria, are affected by gentamicin in proteoglycan-deficient cells. **(A,B)** CHO WT and CHO Δ*XylT* cells were infected at MOI 100 with *S*. Typhimurium [p4889] and then incubated in presence of 100 μg/mL gentamicin. 24 h p.i., cell monolayers were washed, fixed with 4% PFA and stained with DAPI. Microscopy revealed a significantly higher number of GFP-expressing, cytosolic *Salmonella* (indicated by arrows) in CHO Δ*XylT* cells compared to CHO WT. Scale bars, 10 μm. Mann–Whitney *U* test, **p < 0.002. **(C)** CHO WT and CHO Δ*XylT* cells were infected with *S*. Typhimurium WT at MOI 50, and gentamicin (100 μg/mL) was added 30 min after infection. 24 h p.i., cells were treated with 400 μM chloroquine for 1 h, then washed with PBS and lysed. Mann–Whitney *U* test, ***p < 0.001. **(D–E)** CHO WT and CHO Δ*XylT* cells were infected with either *S*. Typhimurium WT, *S*. Typhimurium Δ*sifA*, or *S*. Typhimurium Δ*ssaR* at MOI 50. Gentamicin (100 μg/mL) was added 30 min after infection. 1.5 h p.i. medium was replaced with medium containing gentamicin at 10, 100, or 200 μg/mL as indicated. Intracellular CFU counts were determined 1.5 and 24 h p.i. and depicted is the intracellular replication (CFU ratio of 24 to 1.5 h). **(D)** Cytosolic bacteria (Δ*sifA*) showed higher intracellular replication than WT *Salmonella* in both CHO cell types. Data points, means and SD of representative results of two independent experiments are depicted. Mann–Whitney *U* test, n.s. (not significant), *p < 0.033. **(E)** Intracellular replication of *S*. Typhimurium Δ*ssaR* mutant compared to WT *S*. Typhimurium was increased in CHO WT but not in CHO Δ*XylT* cells. Data points, means and SD of representative results of three independent experiments are depicted. Unpaired *t*-test, n.s. (not significant), ***p < 0.001.

To investigate if a lack of proteoglycans might affect subcellular localization of intracellular bacteria, CHO WT and CHO Δ*XylT* cells were infected with a *S*. Typhimurium reporter strain expressing DsRed protein constitutively and sfGFP only when bacteria are located in the cytosol (31). Microscopy revealed that CHO Δ*XylT* cells incubated with 100 μg/mL gentamicin had significantly lower numbers of total bacteria, but significantly higher numbers of GFP-expressing, cytosolic *Salmonella* compared to CHO WT at 24 h p.i., as shown by an elevated ratio of cytosolic/total bacteria in infected cells (Figures 3A,B). Addition of chloroquine selectively kills *Salmonella* within SCVs (34). Therefore, we used a combination of chloroquine resistance assay and gentamicin protection assay to determine cytosolic and intra-SCV bacteria corroborating our results obtained with the reporter strains (Figure 3C). To further investigate the subcellular localization of *Salmonella*, *S*. Typhimurium Δ*sifA* strain was utilized. This mutant is not able to maintain SCV integrity upon infection, which results in an extensive cytosolic replication of bacteria (30). Intracellular replication was analyzed by gentamicin protection assay, and expressed as a ratio of replicated (CFU at 24 h p.i)/invaded (CFU at 1.5 h p.i) bacteria. Intracellular proliferation of *S*. Typhimurium Δ*sifA* in CHO WT cells in the presence of 100 μg/mL gentamicin was about two times higher when compared to *S*. Typhimurium WT. In contrast, in CHO Δ*XylT* cells, *S*. Typhimurium Δ*sifA* intracellular replication was about 50 times higher compared to *S*. Typhimurium WT strain (Figure 3D). The differences in late replication were even more pronounced in CHO and CHO Δ*XylT* cells incubated with 200 μg/mL gentamicin (Figure 3D). Overall, these data indicate that the reduction of bacterial burden in CHO Δ*XylT* cells was due to a diminished number of bacteria in SCV, while cytosolic bacteria were largely unaffected by increasing concentrations of gentamicin.

Recently, it has been shown that *Salmonella*-induced filaments (SIFs) can increase the exposure of bacteria to internalized antibiotics in the SCV (41). To evaluate a contribution of the SIF network to the observed phenotype, CHO WT and CHO Δ*XylT* cells were infected with either *S*. Typhimurium WT or *S*. Typhimurium Δ*ssaR* (a SPI-2 mutant lacking SIFs) (29). In agreement with the findings by Liss et al. (41), incubation of the infected CHO WT cells with 200 μg/mL gentamicin for 24 h resulted in a significantly higher intracellular proliferation of *S.* Typhimurium Δ*ssaR* compared to the WT strain. In contrast, numbers of both intracellular *Salmonella* WT and Δ*ssaR* were strongly decreased in CHO Δ*XylT* cells (Figure 3E) indicating that the gentamicin-mediated inhibition of bacterial growth in CHO Δ*XylT* cells is independent of SIFs.

### Proteoglycans Are Important for PIKfyve-Dependent Endo-Lysosomal Fusion

**FIGURE 4:**
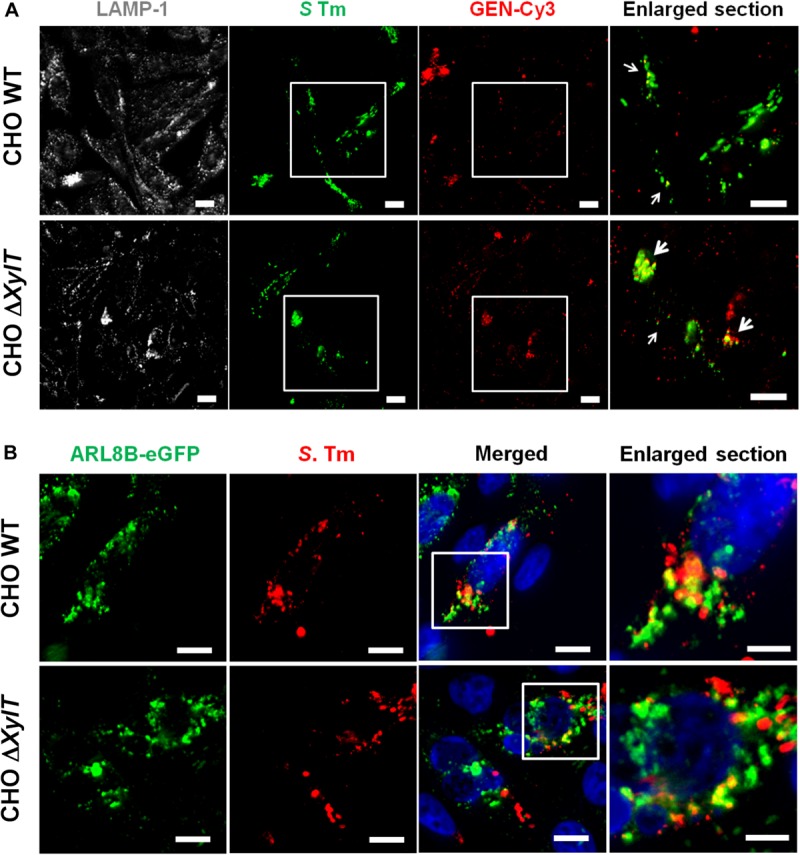
Intracellular gentamicin is associated with *Salmonella* in CHO Δ*XylT* cells. **(A)** CHO cells were infected with *S*. Typhimurium WT, incubated with Cy3-labeled gentamicin 30 min p.i., and 7 h p.i. fixed with 4% PFA, stained with anti-*Salmonella* antibody, Phalloidin-iFluor647 and DAPI. Microscopy revealed an enhanced co-localization of Cy3-labeled gentamicin and *Salmonella* in CHO Δ*XylT* cells (bottom row) resulting in a degradation of bacteria (enlarged section, indicated by arrows). Scale bars, 10 μm. A representative image of three biological repetitions is shown. **(B)** CHO WT and CHO Δ*XylT* cells were transfected with a plasmid encoding the human ARL8B gene fused to eGFP. Cells were infected with *S*. Typhimurium WT at MOI of 50. Eight hours p.i. cells were fixed and stained for *S*. Typhimurium (in red). Representative images of two biological repetitions, scale bars, 10 μm or 5 μm (in enlarged sections).

Our observation that CHO WT and CHO Δ*XylT* cells had similar levels of intracellular gentamicin was based on ELISA measurements of whole cell lysates. Next, we tested whether intracellular localization of gentamicin is altered in the absence of proteoglycans. Indeed, in infected CHO Δ*XylT* cells Cy3-labeled gentamicin was found close to *Salmonella*, or bacterial debris, while in CHO WT cells gentamicin-Cy3 was distributed more randomly (Figure 4A). Of note, uninfected CHO WT and Δ*XylT* cells were similar in terms of a distribution of labeled gentamicin (Supplementary Figure S4). Such re-distribution of an antibiotic may enhance *Salmonella* killing within modified compartments of CHO Δ*XylT* cells. Association of bacteria with vacuolar markers such as LAMP-1 or ARL8B was similar in CHO WT and CHO Δ*XylT* cells (Figure 4B).

**FIGURE 5:**
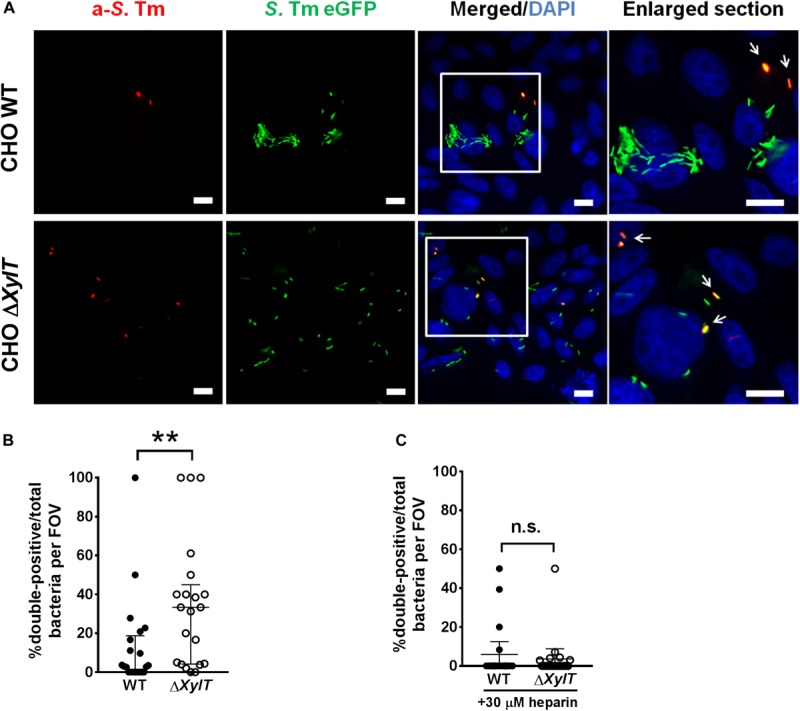
Endocytosed cargo in infected proteoglycan-deficient CHO Δ*XylT* cells co-localizes with *Salmonella*. CHO cells were infected with *S*. Typhimurium eGFP. Gentamicin (100 μg/mL) was added 30 min after infection and 1.5 h p.i. anti-*Salmonella* antibody was added to the medium. 24 h p.i. cells were fixed with 4% PFA, and stained with DAPI. **(A)** Double-positive bacteria are indicated with arrows in enlarged section. Scale bars, 10 μm. **(B)** Number of double-positive bacteria was counted in 20 FOVs. Mann–Whitney *U* test, **p < 0.002. **(C)** 1.5 h p.i. heparin (30 μM) was added to growth medium containing anti-*Salmonella* antibody. 20 random FOVs were counted, mean values with 95% CI are shown. Mann–Whitney *U* test, n.s., not significant.

We hypothesized that a lack of PGs may also alter intracellular routing of cargo other than antibiotics. To test this, we employed an antibody uptake assay. Cells were infected with GFP-expressing *S*. Typhimurium, and an anti-*Salmonella* antibody was added to cell culture medium 1.5 h p.i. (after invasion of bacteria). 24 h p.i., we observed that significantly higher numbers of intracellular *S*. Typhimurium were stained with the anti-*Salmonella* antibody in infected CHO Δ*XylT* cells compared to CHO WT cells (Figures 5A,B). Addition of heparin to the cell culture medium 1.5 h p.i. reduced the number of double-positive bacteria in the infected CHO Δ*XylT* cells, while the total number of *S*. Typhimurium increased. These data indicates an important role of PGs in proper vesicle trafficking (Figure 5C).

**FIGURE 6:**
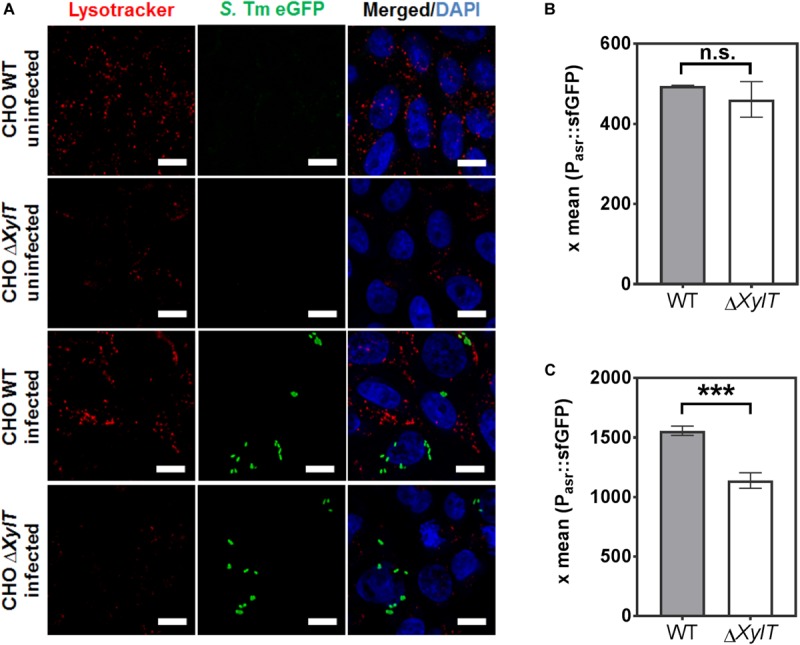
Acidic organelles in CHO Δ*XylT* cells display reduced labeling by Lysotracker. **(A)** Uninfected CHO WT and CHO Δ*XylT* cells, or cells infected with *S*. Typhimurium EGFP at MOI of 50, were incubated for 2 h with 50 nM Lysotracker Red. Cells were fixed with 4% PFA. Representative images of three biological repetitions, scale bars, 10 μm. **(B,C)** Acidification of *Salmonella* is dependent on the presence of PGs. CHO WT or CHO Δ*XylT* cells were infected with *Salmonella* WT harboring an acid shock sensor. Infection and analyses by flow cytometry were performed as described in Supplementary Figure S6. After infection for 30 min, cells were treated with 100 μg/mL gentamicin for 1 h followed by 10 μg/mL gentamicin for 1 h **(B)** or 7 h **(C)**. The mean sfGFP fluorescence intensity is displayed for CHO cells harboring DsRed-positive *Salmonella*. X-means and standard deviations are shown for triplicate samples, and the data shown are representative for three biological replicates with similar outcome. Unpaired *t*-test, n.s., not significant, ***p < 0.001.

Vacuole acidification is sensed and manipulated by *Salmonella*. To test whether endo-lysosomal trafficking and acidic vacuole formation is affected by proteoglycans we stained acidic organelles by incubation with Lysotracker. Strikingly, CHO Δ*XylT* cells (both non-infected and infected) were less stained than CHO WT cells when incubated with Lysotracker (Figure 6A). Interestingly, complemented CHO c*XylT* cells displayed an intermediate degree of Lysotracker staining (Supplementary Figure S5). To test if a lack of PG affects SCV acidification, we used a *Salmonella* strain harboring dual fluorescence reporter p5386 to monitor exposure of intracellular *Salmonella* to acidic pH (Supplementary Figures S6AB). The reporter features constitutive expression of DsRed, allowing the localization of intracellular *Salmonella*, and sfGFP under control of the acid shock response-activated promoter P*_*asr*_* (42, 43). *In vitro* analyses demonstrated the P*_*asr*_* is activated if *Salmonella* is exposed to media of pH 5.0 or lower. Exposure to media with higher pH did not lead to synthesis of sfGFP under control of P*_*asr*_* (Supplementary Figure S6C). Inhibition of acidification of the SCV by addition of vATPase inhibitor bafilomycin fully ablated expression of P*_*asr*_*:sfGFP (Supplementary Figure S6D). Expression of P*_*asr*_*:sfGFP at 2 h p.i. was not affected by presence of absence of gentamicin in the cell culture medium (Figure 6B). However, at 8 h p.i, we observed lower expression of P*_*asr*_*:sfGFP in CHO Δ*XylT* cells compared to CHO WT cells (Figure 6C). Taken together, the acidification of endosomal compartments is impaired in PG-deficient cells as indicated by the lower signal intensity of Lysotracker labeling and lower expression of P*_*asr*_*:sfGFP in Δ*XylT* cells.

**FIGURE 7:**
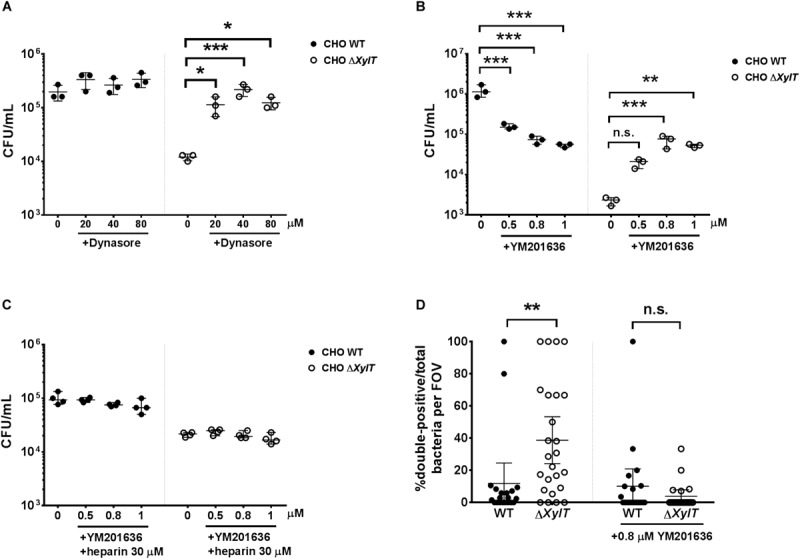
Inhibition of endo-lysosomal fusion increases *Salmonella* burdens in CHO Δ*XylT* cells. CHO WT and CHO Δ*XylT* cells were infected with *S*. Typhimurium WT at MOI = 50 and incubated for 24 h in presence of 100 μg/mL gentamicin and increasing concentrations of **(A)** dynasore or **(B)** YM201636. One-way ANOVA with Dunnett’s multiple comparison test, n.s. (not significant), *p < 0.033, **p < 0.002, ***p < 0.001. **(C)** CHO WT and CHO Δ*XylT* cells were first infected with *S*. Typhimurium WT at MOI of 50. After 30 min, cells were incubated with 100 μg/mL gentamicin, heparin (30 μM), and different concentrations of YM201636 for 24 h. One-way ANOVA with Dunnett’s multiple comparison test, not significant differences are not indicated**. (D)** CHO cells were infected with *S*. Typhimurium eGFP. Gentamicin (100 μg/mL) was added 30 min after infection and 1.5 h p.i. anti-*Salmonella* antibody and 0.8 μM YM201636 were added to the medium. 24 h p.i., cells were fixed with 4% PFA. 20 random FOV were counted. Graph shows mean values with 95% CI. Mann–Whitney *U* test, **p < 0.002, n.s., not significant.

To identify, at which stage trafficking of endocytosed cargo is affected by the lack of PGs, we utilized inhibitors of clathrin-mediated endocytosis (dynasore), phosphoinositide 3-kinase PI3K (wortmannin), as well as an inhibitor of FYVE finger-containing phosphoinositide kinase (PIKfyve) activity (YM201636). Application of dynasore 1.5 h p.i. (added after invasion of bacteria resulted in a significantly higher recovery of *S.* Typhimurium from CHO Δ*XylT* cells compared to the non-treated controls. In contrast, dynasore treatment had no significant effect on intracellular bacterial numbers in CHO WT cells (Figure 7A). In addition, treatment of either CHO WT or CHO Δ*XylT* cells with wortmannin had no significant effect on intracellular *S*. Typhimurium numbers (Supplementary Figure S7). When PIKfyve activity in CHO WT cells was inhibited with YM201636, *S.* Typhimurium numbers were reduced in a dose-dependent manner in agreement with the results by Kerr et al. (44). However, YM201636 treatment of CHO Δ*XylT* cells resulted in significantly more intracellular bacteria compared to non-treated cells (Figure 7B). Heparin treatment abrogated the dose-dependent effect of YM201636 on *Salmonella* survival (Figure 7C) implying a direct effect of PGs on PIKfyve activity. Soluble heparin was detected within endosomal compartments and SCVs in the heparin-treated CHO Δ*XylT* cells as revealed by immunostaining (Supplementary Figure S10). Application of YM201636 also resulted in diminished numbers of double-positive bacteria in both CHO cell lines in an antibody uptake assay (Figure 7D). Furthermore, incubation of CHO WT and CHO Δ*XylT* cells with PIKfyve-inhibitor dramatically enhanced the size of acidic lysosomes (Supplementary Figure S8). These data demonstrate a critical role of proteoglycans in PIKfyve-mediated fusion events.

**FIGURE 8:**
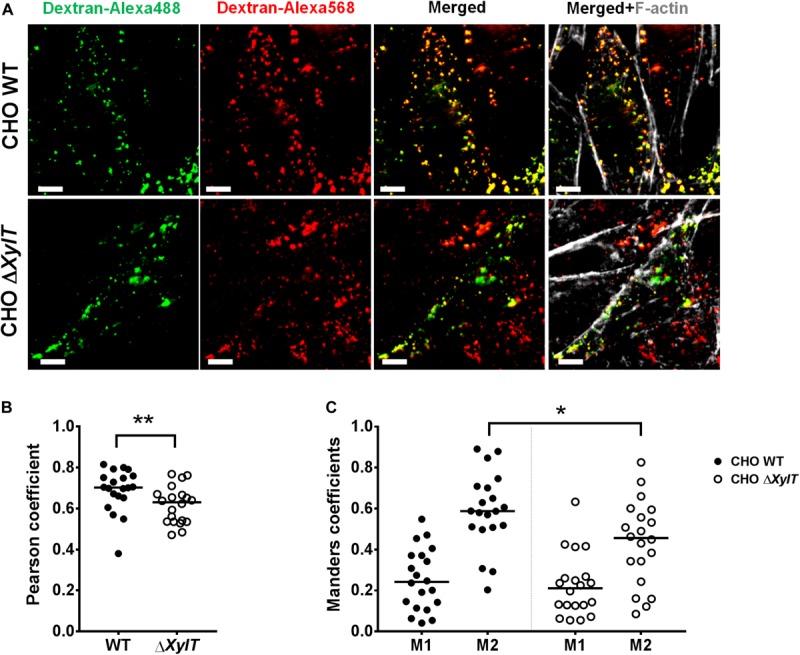
CHO Δ*XylT* cells display reduced degree of endo-lysosomal fusion. CHO WT and CHO Δ*XylT* cells were seeded on cover slips and pulsed for 4 h with dextran-Alexa568, followed by incubation with CHO medium. 18 h later dextran-Alexa488 was added for 10 min, cells were washed and 30 min later, cells were fixed with 4% PFA. **(A)** Co-localization of Alexa-labeled vesicles can be seen in the merged section. Scale bars, 5 μm. **(B)** Pearson correlation coefficient was calculated for 20 FOVs per cell line. **(C)** CHO Δ*XylT* cells have same fraction of late endosomes overlapping with early endosomes (M1), but smaller fraction of early endosomes co-localizing with late endosomes (M2). Mander’s overlap coefficient calculated for each of 20 FOVs, Mann–Whitney *U* test, *p < 0.033, **p < 0.002. Data are representative of two biological repetitions, median values are indicated on graphs.

To assess if endo-lysosomal fusion is abrogated in CHO Δ*XylT* cells, we employed a modified pulse-chase assay using Alexa568- and Alexa488-labeled dextrans to label lysosomes/late endosomes and early endosomes, respectively, as described by Kerr et al. (44). CHO WT and CHO Δ*XylT* cells were incubated with dextran-Alexa568 for 4 h, followed by incubation in dextran-free CHO medium. 18 h later, cells were incubated with dextran-Alexa488 for 10 min, washed and incubated in CHO medium for another 30 min before fixation with 4% PFA. To assess vesicle fusion events, Pearson’s correlation coefficient and Manders split coefficients (M1 and M2, thresholds set manually for both channels) were calculated. CHO Δ*XylT* cells had significantly lower degree of co-localization between the two dextrans compared to CHO WT cells indicating a delayed or reduced endo-lysosomal fusion in proteoglycan-deficient cells (Figure 8).

## Discussion

In the present study, we report a novel role of PGs for endo-lysosomal fusion. PG deficiency abrogates endo-lysosomal fusion which affects *Salmonella* survival within epithelial cells in a context of gentamicin protection assay. Wild-type CHO cells exclusively utilize *Xylt2* for PGs biosynthesis and lack detectable *Xylt1* gene expression (45). CHO pgsA745 (Δ*XylT2*) cells lack the xylosyltransferase-II enzyme and thus, are PG-deficient (3). This cell line has been extensively used to investigate the contribution of PGs to the entry of bacterial and viral pathogens into host cells (46). Recently, it has been identified that CHO Δ*XylT* cells also carry a mutation in the *Lama2* gene. The resulting deletion of the long isoform of the laminin subunit α-2 significantly reduced invasion of group B *Streptococcus* in CHO Δ*XylT* compared to CHO WT cells (47). Indeed, while both the short and the long isoforms of laminin-2 were expressed in our CHO WT cells, CHO Δ*XylT* cells lacked the long isoform expression (Supplementary Figure S9). In addition, it was shown that when *Salmonella* is grown under *pagN*-inducing conditions there was a reduced uptake into CHO Δ*XylT* cells (7). However, in our study, no differences in terms of *S*. Typhimurium adhesion to and invasion into the CHO WT and CHO ΔXylT cell lines were detected.

Upon invasion, *Salmonella* hijack endo-lysosomal trafficking and acquire host factors including LAMP1 and ARL8B in order to establish a *Salmonella*-containing vacuole (SCV) and later on, *Salmonella*–induced filaments (SIFs). Microscopy and chloroquine resistance assays revealed that in the absence of PGs, significantly less bacteria were associated with SCVs when compared to WT CHO cells, while similar numbers of cytosolic bacteria were found in CHO WT and CHO Δ*XylT* cells. It should be noted that no differences in the levels of a total intracellular gentamicin between CHO WT and CHO Δ*XylT* cells were detected. Thus, we reasoned that an intracellular localization of gentamicin might be altered in the infected CHO Δ*XylT* cells, which leads to an increased exposure of specific bacterial populations to the antibiotic. Although cell membranes are generally regarded to be impermeable to gentamicin, aminoglycosides can be transported into epithelial cells via endocytosis-dependent and -independent pathways (48). It was previously reported that endocytosis of gentamicin resulted in its accumulation within lysosomes and in increased lysosomal ROS production in kidney epithelial cells (49). CHO Δ*XylT* cells are not defective in terms of endocytosis/phagocytosis (47), which is supported by our data regarding uptake of gentamicin (Cy3-labeled and by ELISA). However, during infection, we observed an increased co-localization of gentamicin and SCV-associated bacteria in proteoglycan-deficient CHO cells. Indeed, the *S.* Typhimurium Δ*sifA* mutant, which cannot establish a functional SCV and therefore localizes to the cytoplasm (50), was significantly less affected by increasing gentamicin concentrations than WT *Salmonella*, in both CHO cell lines. To conclude, the intracellular re-distribution of gentamicin in proteoglycan-deficient CHO cells was associated with a drastic reduction of *Salmonella* counts.

In addition, we detected increased accumulation of an anti-*Salmonella* antibody in SCV/SIF compartments in CHO Δ*XylT* compared to CHO WT cells. This process could be blocked by addition of heparin to cell culture medium or by inhibiting of PIKfyve activity in CHO Δ*XylT* cells. CHO WT cells treated with a specific PIKfyve inhibitor (YM201636) were characterized by significantly reduced bacterial loads compared to untreated controls which is in line with observations by Kerr et al. (44). In contrast, inhibition of PIKfyve in CHO Δ*XylT* cells increased *S.* Typhimurium counts. PIKfyve is a kinase that converts PtdIns3P into PtdIns(3,5)P_2_. It has been suggested that PIKfyve orchestrates the fusion of *Salmonella* macropinosomes with organelles of the late endosomal/lysosomal system (44), and more recent data link PIKfyve activity with the recycling of tight junction proteins (51) and with a re-distribution of endocytosed cargo from/to lysosomes occurring at late stages of endocytic vacuole maturation (52). As we observed a different distribution of Cy3-labeled gentamicin and endocytosed antibody within the CHO WT and CHO Δ*XylT* cells, we speculated that proteoglycans are required for PIKfyve-dependent endo-lysosomal fusion and subsequent trafficking/recycling pathways. Interestingly, CHO Δ*XylT* cells displayed reduced labeling with Lysotracker, which was increased upon treatment with YM201636. The pH inside the SCV can affect bacterial survival in multiple ways: for example, phagosomal pH in macrophages is important for susceptibility of *S*. Typhimurium to gentamicin (53). In addition, there is evidence that autophagosome-lysosome fusion in CHO cells is affected by the pH in acidic compartments (54). While, in our experiments, early acidification of the SCV in CHO WT and CHO Δ*XylT* cells was similar as determined by acid shock response reporter strains, at the later time points acidification of SCV in CHO Δ*XylT* cells was impaired.

Our results raise the question of specific interactions between phosphoinositides and PGs in the context of infection. For example, it was shown that binding of the transmembrane heparan sulfate PG syndecan-4 to phosphatidylinositol 4,5-bisphosphate (PtdIns(4,5)P_2_) is required for formation of focal adhesions (55). Because phosphoinositides are essential for actin assembly, it is not surprising that *Salmonella* can deplete PtdIns(4,5)P_2_ by the effector SigD which results in membrane fission during bacterial invasion (56). However, the role of PGs in SCV/SIF biogenesis and endo-lysosomal trafficking is less clear. Several studies showed that syndecans, along with the endosomal sorting complex required for transport (ESCRT) proteins, are involved in the formation of multivesicular endosomes or bodies (MVBs) (57, 58). MVBs can fuse with lysosomes or be exported as exosomes. It is known that *Salmonella* disturbs normal endosome to lysosome trafficking, affecting the ESCRT system (23) and exocytosis (16, 17). PtdIns(3,5)P_2_ (hence, PIKfyve) regulates endosomal fission and fusion, and MVB formation (20).

Taken together, our data show that altered routes of endocytosed cargo in PG-deficient epithelial cells interfere with vesicle acidification and *Salmonella*-modulated PIKfyve-dependent fusion to establish a replicative niche and thereby elucidate a novel role of PGs in intracellular vesicle trafficking and SCV formation.

## Data Availability Statement

All datasets generated for this study are included in the article/Supplementary Material.

## Author Contributions

AG, AS, HB, FR, MH, and GG: conceptualization. AG, AO, AS, HB, and LK: investigation. AG, AO, AS, LK, FR, HB, MH, and GG: data analysis. AG, AS, and GG: manuscript writing. AG, AO, AS, HB, FR, LK, MH, and GG: manuscript editing and approval.

## Conflict of Interest

The authors declare that the research was conducted in the absence of any commercial or financial relationships that could be construed as a potential conflict of interest.

## Supplementary Material

FIGURE S1 Intracellular *Salmonella* are sensitive to treatment with ampicillin. **(A)** CHO cells were infected with *S*. Typhimurium 14028s WT at MOI 10, and ampicillin was used instead of gentamicin in a protection assay. 24 h p.i., cells had similar low CFU counts. Dotted line indicates limit of detection, ND – not detected. **(B)** Infected CHO WT and CHO Δ*XylT* cells had comparable levels of released lactate dehydrogenase as measured by LDH assay kit. Dotted line indicates limit of detection. Kruskal–Wallis test with Dunn’s multiple comparison test, n.s. (not significant).

FIGURE S2 CHO WT and Δ*XylT* cells had comparable expression levels of gentamicin transporters genes. Gene expression was measured in CHO WT and CHO Δ*XylT* cells, non-infected and infected with *S*. Typhimurium WT. Control CHO cells were incubated without gentamicin. Gene expression was normalized to *Gapdh* and *Rps9*, and control CHO WT data used as a calibrator. Comparable levels of *Abcc2* **(A)**, *Trpv1* **(B)**, and *Trpv4* **(C)**, expression were observed in both CHO WT and Δ*XylT* cells, regardless infected or not. One-way ANOVA with Tukey’s multiple comparison test and Mann-Whitney test, not significant differences are not indicated.

FIGURE S3 Presence of PGs in CHO cells. **(A)** CHO WT, CHO Δ*XylT*, and complemented CHO Δ*XylT* cells (indicated as CHO cXylT) were stained with heparan sulfate-specific antibody and analyzed by flow cytometry. **(B)** *S.* Typhimurium WT was grown for 6 h in LB broth in presence/absence of 30 μM heparin, and OD600 was recorded every 10 min. Mean values of three technical replicates of a representative experiment out of two are shown.

FIGURE S4 Gentamicin uptake in CHO WT and CHO Δ*XylT* cells. CHO cells were incubated for 7 h with gentamicin-Cy3 conjugate and then fixed with 4% PFA. Microscopy revealed a similar distribution of the labeled antibiotic within the CHO WT and CHO Δ*XylT* cells.

FIGURE S5 Labeling by Lysotracker correlates with PGs expression in CHO cells. CHO WT, CHO Δ*XylT*, and complemented CHO c*XylT* cells were infected with *S*. Typhimurium EGFP at MOI 50 and incubated for 24 h with 50 nM Lysotracker Red added upon invasion of bacteria. CHO c*XylT* cells had an intermediate staining (compare to Supplementary Figure S3A). Representative images of two biological repetitions. Scale bars, 10 μm.

FIGURE S6 A dual fluorescence reporter for acid shock exposure of *Salmonella*. **(A)** Plasmid map of p5386, encoding DsRed constitutively under control of promoter P_*EM7*_, and sfGFP under control the acid shock-inducible promoter P*_*asr*_*. **(B)** Flow cytometry and gating of *Salmonella* without fluorescent protein expression, or constitutive expression of DsRed or sfGFP. **(C)** Acid shock of cultured bacteria induces sfGFP expression. *Salmonella* WT harboring p5386 (*S*.Tm WT) was grown in PCN, pH 7.5 to mid-log phase. Bacteria were pelleted, washed twice in sterile saline, and resuspended in PCN buffered to the indicated pH. Culture was continued for 1 h, bacteria were harvested by centrifugation and resuspended in PCB containing 200 μg/mL chloramphenicol to stop further protein biosynthesis. The bacteria were incubated for at least 2 h at 4°C to allow full maturation of sfGFP, and subjected to flow cytometry. **(D)** For *in vivo* analyses, *S*.Tm WT harboring p5386 was subcultured for 3 h and used to infect ca. 2 × 10^5^ CHO WT or CHO Δ*XylT* cells at MOI of 10. If indicated (+ Baf), bafilomycin was added to a final concentration of 100 nM. Cells were infected for 30 min, washed three times to remove non-internalized bacteria and incubated 2 h with or without gentamicin addition as indicated in Fig 6B. A representative example of an assay with a constant concentration of 10 μg/mL gentamicin is shown. After washing, cells were detached using biotase, chloramphenicol was added to final concentration of 200 μg/mL and incubated for at least 4 h at 4°C for allow full maturation of sfGFP. Flow cytometry was performed by gating of CHO cells and the level of DsRed and sfGFP fluorescence was determined for at least 50,000 infected host cells.

FIGURE S7 Inhibition of PI3K does not affect intracellular *Salmonella*. CHO WT and CHO Δ*XylT* cells were infected with *S*. Typhimurium WT at MOI of 50 and incubated for 24 h with 100 μg/mL gentamicin and increasing concentrations of specific inhibitors. Addition of wortmannin had no effect on *Salmonella* survival in either CHO cell line. One-way ANOVA with Dunnett’s multiple comparison test, only significant differences are indicated.

FIGURE S8 PIKfyve kinase inhibition increased labeling by Lysotracker in both CHO WT and CHO Δ*XylT* cells. CHO cells infected with *S*. Typhimurium EGFP were incubated for 24 h with 50 nM Lysotracker Red added upon invasion, in presence/absence of 0.8 μM YM201636. Microscopy revealed enlarged lysosomes/endosomes in YM201636-treated CHO cells. Representative images, scale bars, 10 μm.

FIGURE S9 CHO Δ*XylT* cells lack *Lama2* expression. cDNA of the uninfected CHO cells was used to screen for an expression of *Lama2* isoforms. ND – not detected.

FIGURE S10 Addition of heparin to the medium results in intracellular accumulation of heparin in the endo-lysosomal system. CHO Δ*XylT* cells, uninfected and infected with *S*. Typhimurium WT at MOI of 50 were incubated for 24 h with 100 μg/mL gentamicin and with 30 μM heparin. Heparin (green) was detected inside CHO Δ*XylT* cells, in the same compartment as bacteria (red). In CHO WT cells, in the absence of added heparin, HS staining shows localization of HS at the cell surface but also in endo-lysosomal compartments. Representative images of two biological repetitions, scale bars, 10 μm or 5 μm (in enlarged sections).

TABLE S1 Primers used in this study.

TABLE S2 Antibodies used in this study.
