# Supplementary figures and images for: Proteoglycan-Dependent Endo-Lysosomal Fusion Affects Intracellular Survival of Salmonella Typhimurium in Epithelial Cells
Source: Front Immunol. 2020 Apr 29;11:731. doi: 10.3389/fimmu.2020.00731 (PMC7201003; doi:10.3389/fimmu.2020.00731)

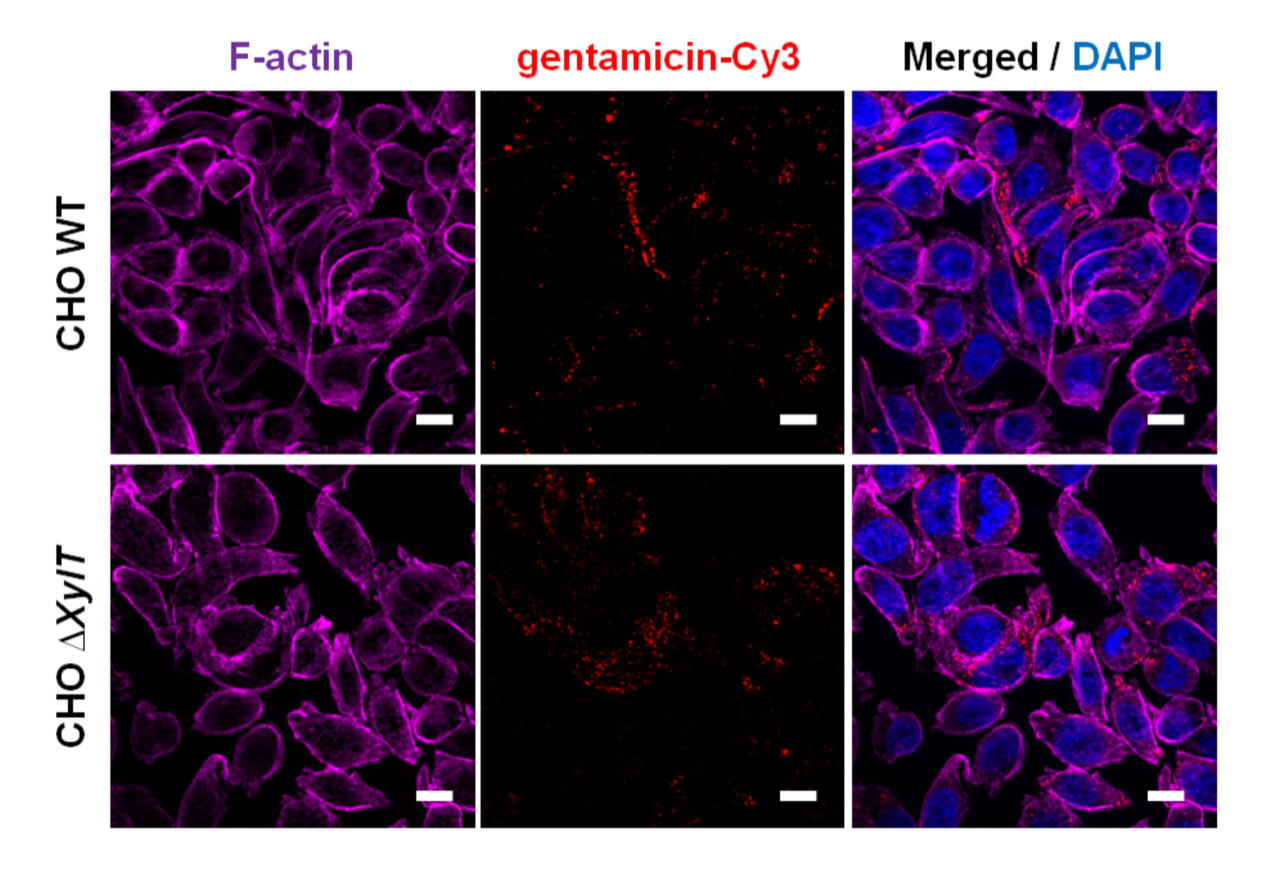

Supplement: FIGURE S4 — Gentamicin uptake in CHO WT and CHO ΔXylT cells. CHO cells were incubated for 7 h with gentamicin-Cy3 conjugate and then fixed with 4% PFA. Microscopy revealed a similar distribution of the labeled antibiotic within the CHO WT and CHO ΔXylT cells. [file Image_4.TIF]

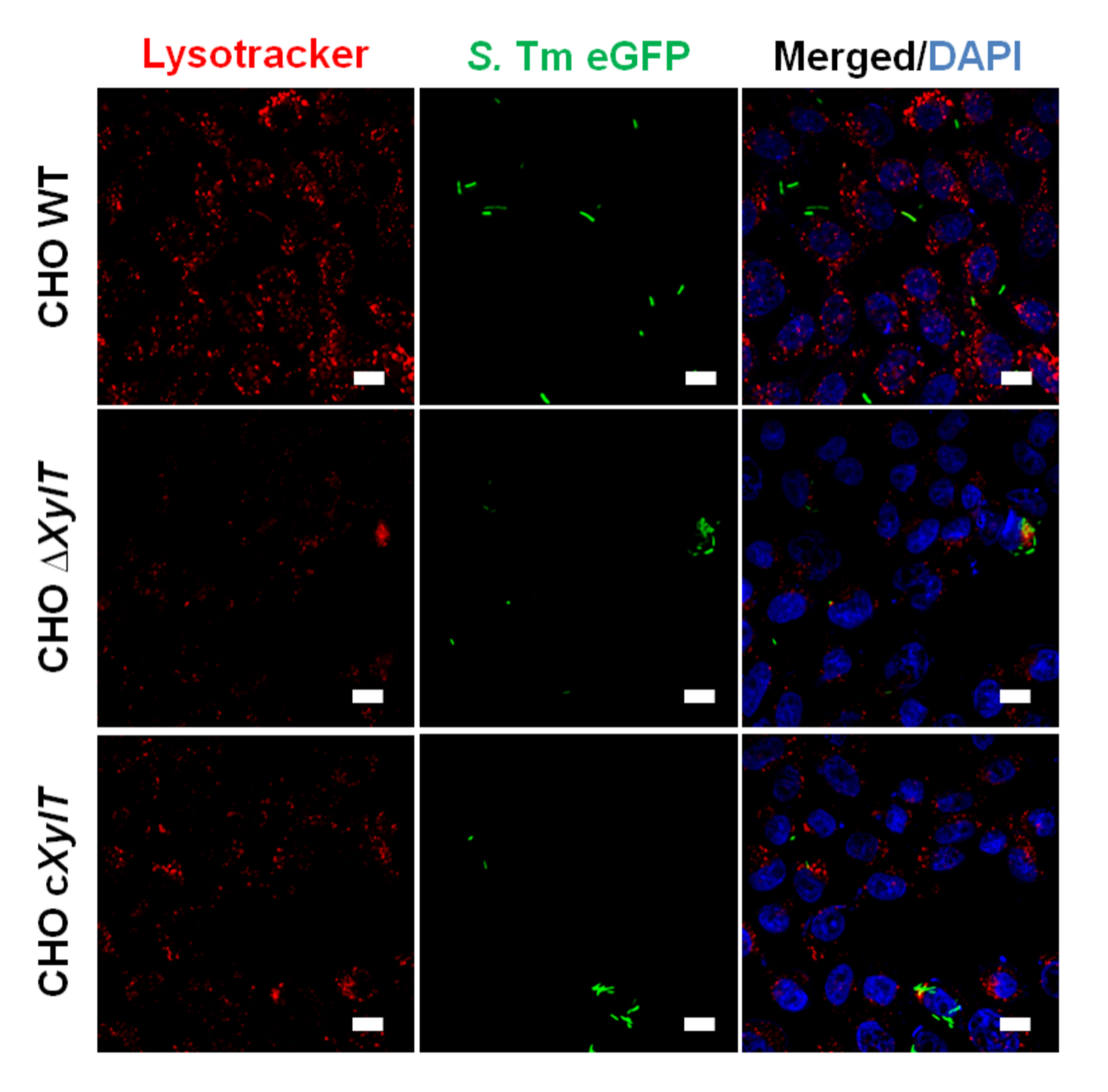

Supplement: FIGURE S5 — Labeling by Lysotracker correlates with PGs expression in CHO cells. CHO WT, CHO ΔXylT, and complemented CHO cXylT cells were infected with S. Typhimurium EGFP at MOI 50 and incubated for 24 h with 50 nM Lysotracker Red added upon invasion of bacteria. CHO cXylT cells had an intermediate staining (compare to Supplementary Figure S3A). Representative images of two biological repetitions. Scale bars, 10 μm. [file Image_5.TIF]

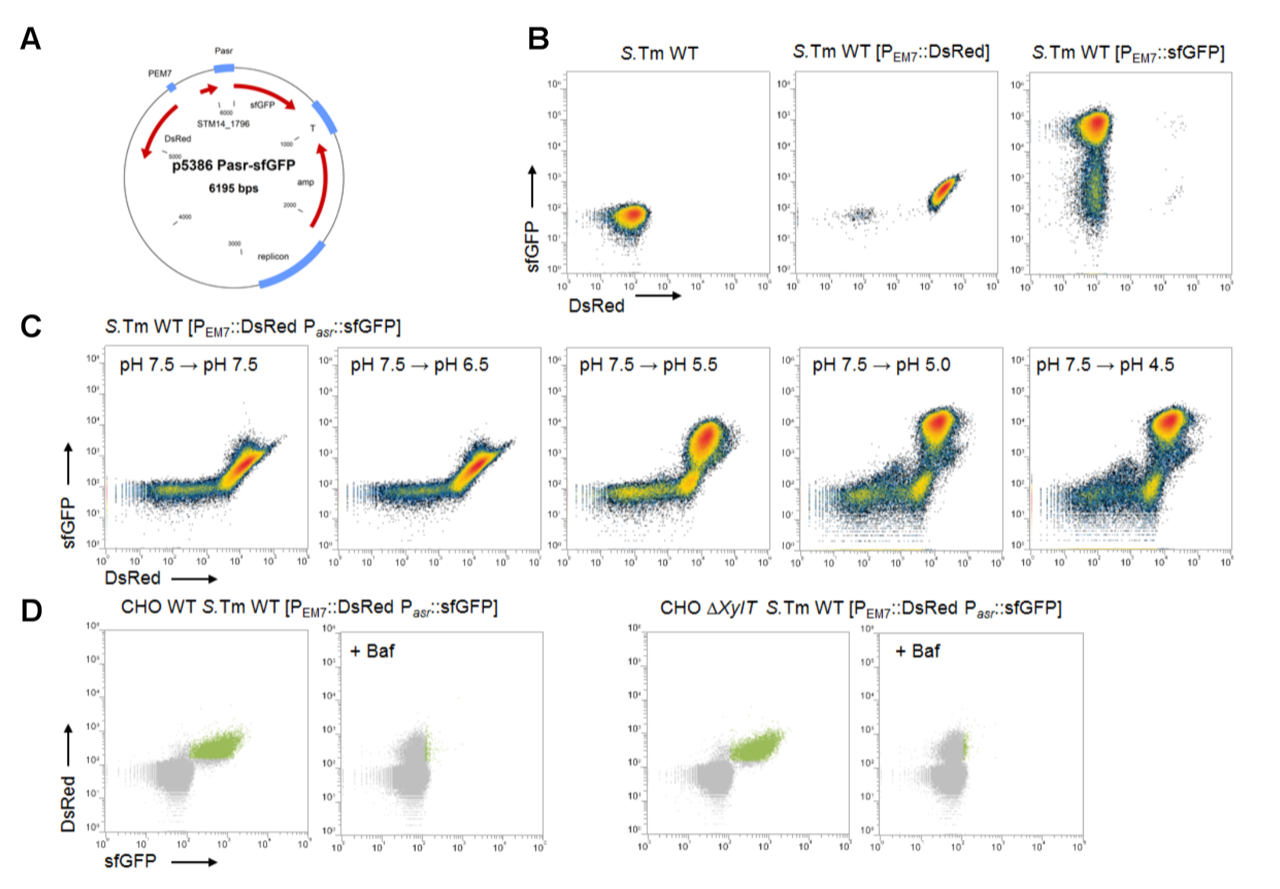

Supplement: FIGURE S6 — A dual fluorescence reporter for acid shock exposure of Salmonella. (A) Plasmid map of p5386, encoding DsRed constitutively under control of promoter PEM7, and sfGFP under control the acid shock-inducible promoter Pasr. (B) Flow cytometry and gating of Salmonella without fluorescent protein expression, or constitutive expression of DsRed or sfGFP. (C) Acid shock of cultured bacteria induces sfGFP expression. Salmonella WT harboring p5386 (S.Tm WT) was grown in PCN, pH 7.5 to mid-log phase. Bacteria were pelleted, washed twice in sterile saline, and resuspended in PCN buffered to the indicated pH. Culture was continued for 1 h, bacteria were harvested by centrifugation and resuspended in PCB containing 200 μg/mL chloramphenicol to stop further protein biosynthesis. The bacteria were incubated for at least 2 h at 4°C to allow full maturation of sfGFP, and subjected to flow cytometry. (D) For in vivo analyses, S.Tm WT harboring p5386 was subcultured for 3 h and used to infect ca. 2 × 10^5^ CHO WT or CHO ΔXylT cells at MOI of 10. If indicated (+ Baf), bafilomycin was added to a final concentration of 100 nM. Cells were infected for 30 min, washed three times to remove non-internalized bacteria and incubated 2 h with or without gentamicin addition as indicated in Fig 6B. A representative example of an assay with a constant concentration of 10 μg/mL gentamicin is shown. After washing, cells were detached using biotase, chloramphenicol was added to final concentration of 200 μg/mL and incubated for at least 4 h at 4°C for allow full maturation of sfGFP. Flow cytometry was performed by gating of CHO cells and the level of DsRed and sfGFP fluorescence was determined for at least 50,000 infected host cells. [file Image_6.TIF]

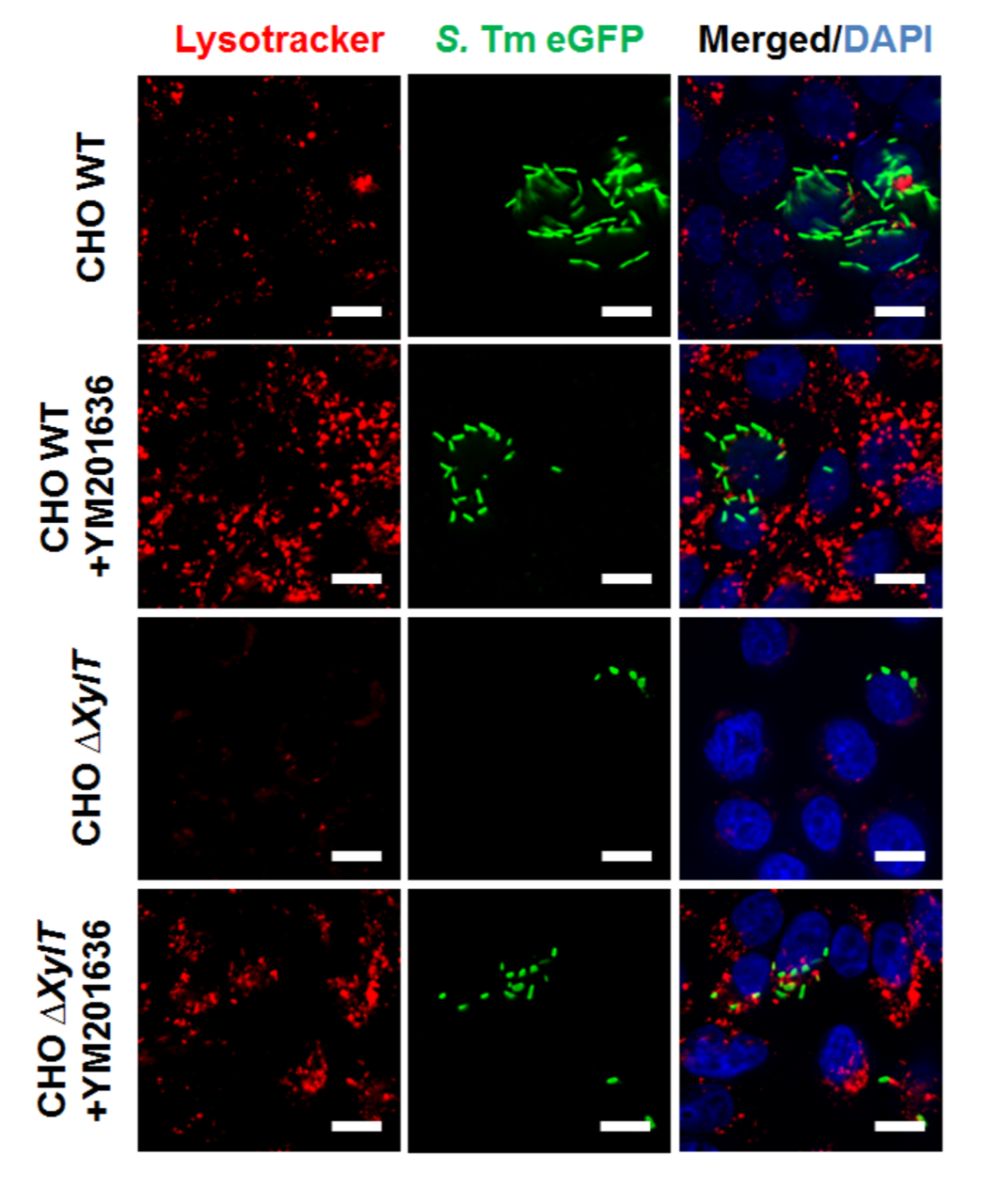

Supplement: FIGURE S8 — PIKfyve kinase inhibition increased labeling by Lysotracker in both CHO WT and CHO ΔXylT cells. CHO cells infected with S. Typhimurium EGFP were incubated for 24 h with 50 nM Lysotracker Red added upon invasion, in presence/absence of 0.8 μM YM201636. Microscopy revealed enlarged lysosomes/endosomes in YM201636-treated CHO cells. Representative images, scale bars, 10 μm. [file Image_8.TIF]

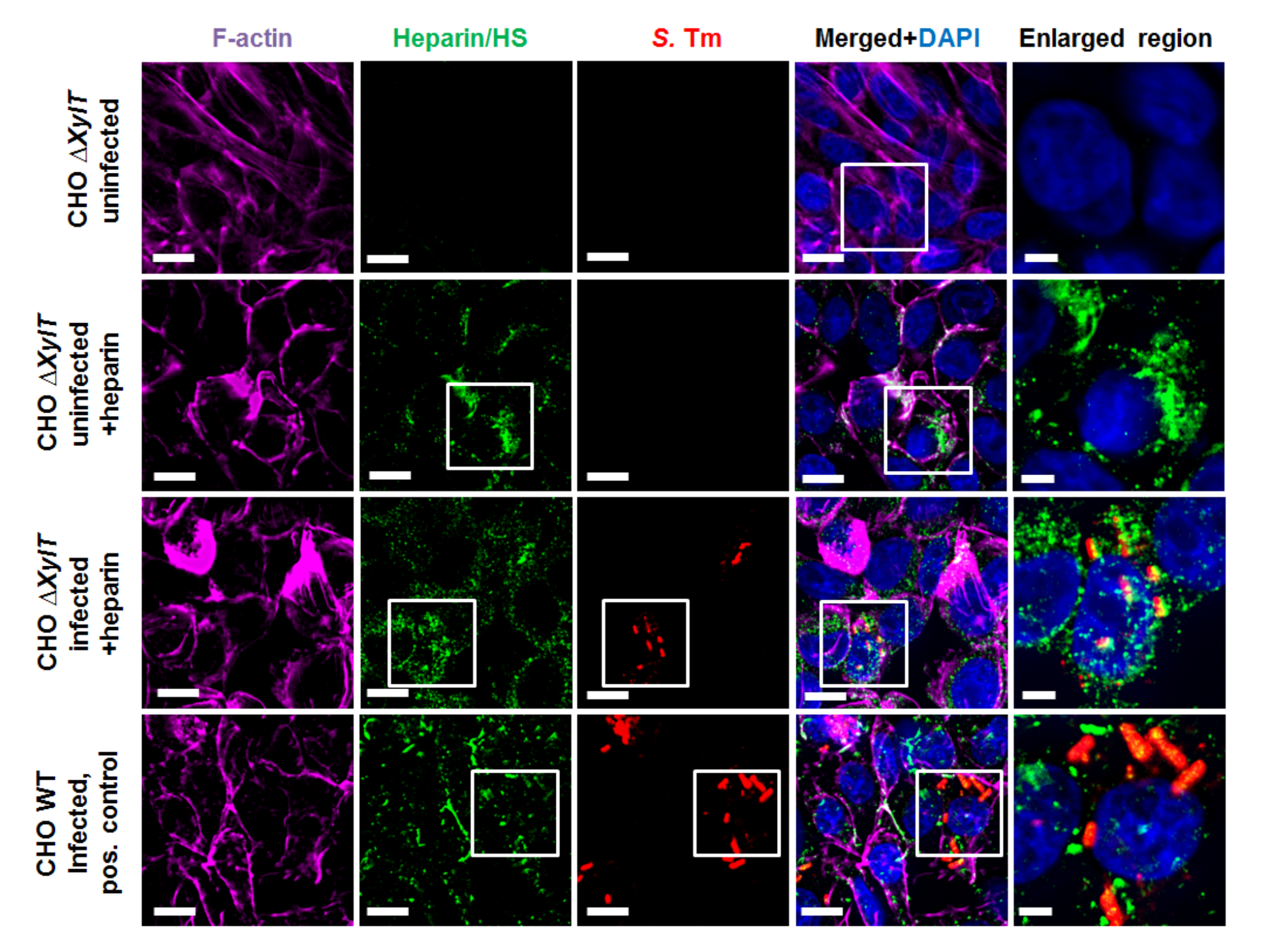

Supplement: FIGURE S10 — Addition of heparin to the medium results in intracellular accumulation of heparin in the endo-lysosomal system. CHO ΔXylT cells, uninfected and infected with S. Typhimurium WT at MOI of 50 were incubated for 24 h with 100 μg/mL gentamicin and with 30 μM heparin. Heparin (green) was detected inside CHO ΔXylT cells, in the same compartment as bacteria (red). In CHO WT cells, in the absence of added heparin, HS staining shows localization of HS at the cell surface but also in endo-lysosomal compartments. Representative images of two biological repetitions, scale bars, 10 μm or 5 μm (in enlarged sections). [file Image_10.TIF]
