# Supplementary material for: Proteoglycan-Dependent Endo-Lysosomal Fusion Affects Intracellular Survival of Salmonella Typhimurium in Epithelial Cells
Source: Front Immunol. 2020 Apr 29;11:731. doi: 10.3389/fimmu.2020.00731 (PMC7201003; doi:10.3389/fimmu.2020.00731)
Supplement: TABLE S1 — Primers used in this study. [file Table_1.DOCX]

**Table S1. Primers used in this study.**

| **Gene/Primer name** | **Sequence** |
| --- | --- |
| *Gapdh* | Fw: CGTATTGGACGCCTGGTTAC  Rv: GGCAACAACTTCCACTTTGC |
| *Rps9* | Fw: TTTACCCTGGCCAAGATCC  Rv: TGGGTCCTTTTCGTCCAG |
| *Trpv1* | Fw: GGTGCTACATGCCCTGGT  Rv: GTCACGAACTTGGTGTTGTCA |
| *Trpv4* | Fw: GAAGGTGTGTGACGAGGATG  Rv: GATGGTGAGCTTGAACAGGTC |
| *Abcc2* | Fw: CACATGGCTCCTGGTGTTAG  Rv: ACTCAGAAATGAAGCCGTGG |
| Vf-p4889  Vr-p4889 | Fw: ATGCGCAAAGGCGAAGAACTGTTTACCGGTGT  Rv: GGCCGGCATCACCGGCGCCACAG |
| 1f Pasr-sfGFP  1r Pasr-sfGFP | Fw: GGCGCCGGTGATGCCGGCCCACGCCTGAAAAGAAATAATCC  Rv: GTTCTTCGCCTTTGCGCATTTTGATACCCTCGATTTGGTTTTC |

**Table S2. Antibodies used in this study.**

| **Antibody** | **Reference/Manufacturer, RRID and catalog number** |
| --- | --- |
| Mouse Anti-*Salmonella* Typhimurium-LPS Monoclonal Antibody, Unconjugated, Clone 1E6 | Meridian Life Science Cat# C86309M, RRID:AB_152809 |
| Rabbit *Salmonella* O Antiserum Group B Factors 1, 4, 12, 27 | BD Difco Cat# 229731 |
| F(ab)2-Goat anti-Rabbit IgG (H+L) Cross-Adsorbed Secondary Antibody, Alexa Fluor 546 | Thermo Fisher Scientific Cat# A-11071, RRID:AB_2534115 |
| F(ab)2-Goat anti-Mouse IgG (H+L) Cross-Adsorbed Secondary Antibody, Alexa Fluor 488 | Thermo Fisher Scientific Cat# A-11017, RRID:AB_2534084 |
| AO4B08, Anti-heparan sulfate Phage Display Antibody with VSV tag | Dam *et al*., 2003 |
| Mouse Anti-VSV-G tag Monoclonal Antibody, Unconjugated, Clone P5D4 | Abcam Cat# ab50549, RRID:AB_883494 |
